# Influence of well-being and quality of work-life on quality of care among healthcare professionals in southwest, Nigeria

**DOI:** 10.1038/s41598-022-25057-w

**Published:** 2023-05-15

**Authors:** Adesola C. Odole, Michael O. Ogunlana, Nse A. Odunaiya, Olufemi O. Oyewole, Chidozie E. Mbada, Ogochukwu K. Onyeso, Ayomikun F. Ayodeji, Opeyemi M. Adegoke, Iyanuoluwa Odole, Comfort T. Sanuade, Moyosooreoluwa E. Odole, Oluwagbohunmi A. Awosoga

**Affiliations:** 1 Department of Physiotherapy, College of Medicine, University of Ibadan, Ibadan, Nigeria; 2 Department of Physiotherapy, Federal Medical Centre, Abeokuta, Nigeria; 3 College of Health Sciences, University of KwaZulu-Natal, Private Bag X54001, Durban, South Africa; 4 Department of Physiotherapy, Olabisi Onabanjo University Teaching Hospital, Sagamu, Ogun State Nigeria; 5 Department of Medical Rehabilitation, Obafemi Awolowo University, Ile-Ife, Nigeria; 6 Faculty of Health Sciences, University of Lethbridge, Alberta, Canada; 7 University College Hospital, Ibadan, Nigeria; 8 Department of Health, Kinesiology, and Applied Physiology, Concordia University, Montreal, Canada; 9 Jericho Nursing Home, Ibadan, Nigeria; 10 Department of Public Health, Yale University, New Haven, CT USA

**Keywords:** Health care, Health occupations

## Abstract

The Nigerian healthcare industry is bedevilled with infrastructural dilapidations and a dysfunctional healthcare system. This study investigated the influence of healthcare professionals’ well-being and quality of work-life (QoWL) on the quality of care (QoC) of patients in Nigeria. A multicentre cross-sectional study was conducted at four tertiary healthcare institutions in southwest, Nigeria. Participants’ demographic information, well-being, quality of life (QoL), QoWL, and QoC were obtained using four standardised questionnaires. Data were summarised using descriptive statistics. Inferential statistics included Chi-square, Pearson’s correlation, independent samples t-test, confirmatory factor analyses and structural equation model. Medical practitioners (n = 609) and nurses (n = 570) constituted 74.6% of all the healthcare professionals with physiotherapists, pharmacists, and medical laboratory scientists constituting 25.4%. The mean (SD) participants’ well-being = 71.65% (14.65), QoL = 61.8% (21.31), QoWL = 65.73% (10.52) and QoC = 70.14% (12.77). Participants’ QoL had a significant negative correlation with QoC while well-being and quality of work-life had a significant positive correlation with QoC. We concluded that healthcare professionals’ well-being and QoWL are important factors that influence the QoC rendered to patients. Healthcare policymakers in Nigeria should ensure improved work-related factors and the well-being of healthcare professionals to ensure good QoC for patients.

## Introduction

Healthcare workers are people engaged in actions with the primary intent of improving health^1^, both in the health and non-health sectors. Health service providers and health management/support employees are the two types of health workers. Professionals (e.g., doctors, nurses, physiotherapists, medical laboratory scientists, and pharmacists), associates (e.g., laboratory technicians), and other community members (e.g., traditional healers) are classified as health service providers. Other professionals (e.g., accountants in the hospital), associates (e.g., administrative staff in the hospital), support staff (e.g., clerical workers and drivers in the hospital), and craft/trade workers (e.g., painters in the hospital) are all examples of health management/support personnel. Healthcare professionals (HCPs) provide varied services owing to the professional heterogeneity of the group as well as the diversity of patients’ needs^2^. There are more than 59 million health workers worldwide, distributed unequally between and within countries, and they are found predominantly in richer areas where health needs are less severe^1^. The demographic characteristics of HCPs differ across regions, with physicians and nurses accounting for the largest group of HCPs globally^3,4^. However, the population of HCPs globally remains woefully insufficient to meet health needs, with the total shortage being in the order of 4.3 million workers^1^.

In Nigeria, female nurses account for most HCPs, and the ratio of HCPs to patients is extremely low across board, with an average of 1.95 HCPs per 1000 people^5^. This is due to complex socio-political and economic problems that reinforce the propensity of caregivers to emigrate to developed countries such as the United States of America, Canada, and the United Kingdom, in search of better well-being^6^. Low health professional-patient ratio leads to a higher workload, work stress, frustration, burnout, job dissatisfaction and absenteeism^7,8^. Inequitable distribution of the health workforce, conflicts among different health professional groups, poor remuneration, and poor welfare of the health workforce have been reported as perhaps the most significant constraint to the development and sustainability of the health system, particularly in Nigeria^5,9^. Consequently, HCPs have begun to migrate in quest of better well-being, quality of life, and quality of work life. According to a report from the World Health Organisation^1^, health workers in Sub-Saharan Africa, including Nigeria, are constantly relocating due to an unfavourable work environment and poor quality of work-life, which has an impact on their well-being.

Well-being, as a spectrum, entails a flourishing, happy, high well-being at one end, and depression, anxiety, and low well-being at the other end^10^. The well-being construct is multifaceted, consisting of emotional well-being, vitality, satisfying life, self-esteem, resilience, and positive functioning^11^. The major factors that influence the well-being of HCPs are working conditions, remuneration, job security, interactions, and interpersonal relationship with colleagues^12–14^. The well-being of HCPs leads to more productivity, optimum job commitment, and delivery of quality care to patients^14,15^. When well-being at work is the construct of interest, quality of work-life (QoWL) is the term used. Quality of work-life is an integral part of quality of life that entails a broader and wider scope than job satisfaction and considers how an employee would evaluate their work environment^16^. Researchers have studied the diverse factors that impact the quality of work-life of HCPs which include workload, staffing, professional autonomy, job satisfaction, and staff welfare^17,18^. Good quality of work-life among HCPs elicits compassion and satisfaction, which has an impact on the quality of care provided to patients^19^. The World Health Organisation^20^ advocates for patients to receive high-quality care that is effective, safe, patient-centred, timely, equitable, inclusive, and efficient. In addition, patient preference is an important factor to consider in delivering good quality of care to patients^21^. To achieve this, healthcare systems ought to provide good working conditions for HCPs to increase their quality of work-life and service delivery^7,22^.

In Nigeria, few studies have been conducted on well-being and quality of work-life among HCPs^23,24^. To our knowledge, this is the first multicentre study in Nigeria investigating the influence of HCPs’ well-being and quality of work-life on quality of care in Nigeria. This study was therefore aimed at investigating the influence of HCPs’ well-being and quality of work-life on the quality of care given to patients in Nigeria. We hypothesised that well-being and quality of work-life will influence HCPs and the quality of care rendered to patients.

## Methods

### Study design

The study was a multicentre cross-sectional survey. Participants’ demographic characteristics, well-being, quality of life, quality of work-life, and quality of care were obtained using four standardised questionnaires.

### Study locations

Southwestern Nigeria is made up of six states. We purposively selected four publicly funded tertiary hospitals in Ogun, Osun, and Oyo states based on the socio-cultural, environmental, political, and socioeconomic similarities of those states. It was assumed that the three states were representative of the others. The selection criteria for the hospitals were: (i) being publicly funded, (ii) being a referral centre with a tertiary level of care, and (iii) having a bed capacity greater than 500. The included hospitals were FMCA and OOUTH Sagamu, in Ogun State; OAUTH, Ile-Ife, Osun State; and UCH Ibadan, Oyo State.

### Study participants

The designated HCPs were medical practitioners (physicians, surgeons, and dentists), nurses, physiotherapists, pharmacists and medical laboratory scientists. Participants were proportionally recruited based on the population size of each hospital using the formula: ([*Z*^2^P{1 – P}]/*e*^2^)/(1 + ([*Z*^2^P{1 – P}]/*e*^2^*N*)), where proportion (P) = 0.5, margin of error (e) = 0.05, Z-score = 1.96, populations (N) = 566 (OOUTH), = 2000 (FMCA), = 3000 (UCH), and 1490 (OAUTH). Therefore, the minimum sample size for OOUTH, FMCA, UCH and OAUTH were 229, 323, 341, and 306, respectively, giving a total of 1199. In anticipation of incomplete survey responses, 1600 participants were recruited.

### Study instruments

A biodata form and four standardised questionnaires were used for data collection. The biodata form was used to obtain information on participants’ demographic variables such as gender, age, years of practice, highest educational qualification, designation, appointment type, work schedule, average weekly work hours, and practice location. Participants’ well-being was assessed using the WHO-5 well-being index and the personal well-being index (PWI) scale^25,26^. The WHO-5 is a five-item questionnaire that assesses participants’ feelings about components of healthy living in the past 2 weeks on a 6-points Likert scale (score 0 to 5). Each participant’s responses were summed (range = 0 to 25) and converted into percentage scores. The WHO-5 has been reported to be valid and reliable in a systematic review of 213 studies that applied the instrument in diverse settings, with average sensitivity and specificity of 0.86 and 0.75, respectively^26^. The PWI is an eight-item valid and reliable questionnaire that assesses participants’ satisfaction with their life, health, life achievements, relationships, safety, community, future security, and spirituality on an 11-points (score 0 to 10) Likert scale^25^. Each participant’s responses were summed and converted into percentage scores^25^. The PWI has been reported to have good psychometric properties: validity, reliability, and sensitivity^27^. The internal consistency measured with Cronbach’s alpha ranges between 0.86 and 0.89^28^. The work-related quality of life (WRQoL) questionnaire was used to assess participants’ quality of work-life. The 24-item questionnaire contains six domains: general well-being, home-work interface, job-career satisfaction, control at work, working conditions, and stress at work^29^. The WRQoL contains 21 positively worded questions and three negatively worded questions (items 7, 9, and 19). The questionnaire asked the extent to which a participant agreed to each of the items as it related to their work-life on a 5-point Likert scale, 1 = strongly disagree to 5 = strongly agree. The WRQoL questionnaire was found valid, reliable, and consistent among a cohort of HCPs, overall scale reliability was 0.91 with good subscale reliabilities ranging from 0.76 to 0.91^17^. Furthermore, the participants’ quality of care was obtained using the Quality of Care (QoC) questionnaire^30^. The 22-item questionnaire was subdivided into two domains: positively worded person-centred care (12 items) and negatively worded discordant care (10 items). The questionnaire inquired on how frequently the listed items had occurred while the participants related with patients in the last 6 months. Responses were on a 6-point Likert scale, 0 = never to 5 = always. The QoC questionnaire has been shown to be valid and reliable, with Cronbach’s alpha of 0.86 and 0.74 for the person-centred care and discordant care subscales respectively.

### Procedure for data collection and storage

Ethical approval was independently obtained from the health research ethics committees of The University of Lethbridge, Alberta, Canada (protocol number 2021-053); Federal Medical Centre, Abeokuta, Ogun State, Nigeria (FMCA/470/HREC/01/2021/07); Olabisi Onabanjo University Teaching Hospital, Sagamu, Ogun State, Nigeria (OOUTH/HREC/415/2021AP); University of Ibadan/University College Hospital, Ibadan, Oyo State, Nigeria (UCH-UI/EC/21/0125); and Obafemi Awolowo University Teaching Hospital Complex, Ile-Ife, Osun State, Nigeria (ERC/2021/11/03). The research was conducted in accordance with the ethical principles guiding human subjects’ research and informed consent was obtained from all participants. The questionnaires were distributed to HCPs within the selected hospitals with the help of research assistants. Healthcare professionals (HCPs) were included in the study if they had worked in one of the selected facilities for at least 6 months and were willing to read and sign a written informed consent form before responding to the survey. The questionnaire was self-administered and returned to research assistants after completion. The primary investigator’s number was boldly printed on the survey to facilitate the return of questionnaires in the case of participants who failed to submit theirs immediately. Data were extracted from the questionnaires and transferred to already coded SPSS spreadsheet in designated computers at the four study locations. Individual datasets were merged into a final anonymised dataset, password encrypted and saved to the cloud.

### Data analyses

Data were analysed using SPSS 27.0 version software (SPSS Inc., Chicago, Illinois, USA). The dataset was cleaned of missing variables, and all entries with more than 25% missing variables in a domain were deleted for the domain. The data were summarised using descriptive statistics: frequency (percentage) and mean (standard deviation). Participants’ well-being, QoWL, and QoC scores were summated in separate columns and converted to percentage points, this was in line with the rubric provided by the instrument developers and the general approach for analysis of Likert scale data^31^. We obtained continuous normally distributed variables for each of the outcomes (skewness < 3.29). We dichotomised the QoC scores into poor and good QoC using a 75% cut-off for good QoC. Inferential statistics included the Chi-square test for differences in the QoC levels across the demographic characteristics; Pearson’s correlation among the outcomes; independent samples t-test analysis for differences in the mean well-being index and QoWL among people classified to have provided poor and good QoC. The domain validity and reliability of the aggregate instrument were obtained using Cronbach alpha and Intraclass correlational statistics. We completed a Confirmatory Factor Analysis (CFA) using Maximum Likelihood with Varimax-orthogonal rotation to determine the new domain membership of the items in the aggregate questionnaire. A structural equation modelling through path analysis for the relationship between QoC with other study outcomes was completed using SPSS Analysis of Moment Structures (AMOS). A maximum likelihood estimation procedure was used to estimate the coefficients. The model fitting was assessed using a Chi-square goodness of fit test as well as the comparative fit index (CFI), the root-mean-square error of approximation (RMSEA), and Tucker-Lewis index (TLI). The measurement of association was interpreted under the standardised mode, using the odds ratio and confidence intervals of 95%.

## Results

### Demographic characteristics of participants

A total of 1600 questionnaires were administered across the four centres. Most of the participants (n = 1580, 98.75% response rate) completed and returned valid surveys which were analysed. The participants’ demographic characteristics are shown in Tables 1 and 2. Many of the participants 1380 (87.3%) were full-time professionals, 1076 (68.1%) held entry-level bachelor’s degrees, 982 (62.2%) were women, and 985 (62.3%) were within the age range of 30 to 49 years. Most of the participants were within the first decade of their appointment (n = 1103, 69.8%), which implies that without recourse to their biological age, many participants have about 25 more service years. Medical practitioners (n = 609) and nurses (n = 570) constituted 74.6% of all the HCPs. The mean (SD) participants’ PWI = 71.65% (14.65), QoL = 61.8% (21.31), QoWL = 65.73% (10.52) and QoC = 70.14% (12.77).

**Table 1:** Participants’ demographic characteristics (n = 1580).

Parameter	n	Quality of care *f* (%)		*df*	χ^2^-statistic	*p*-value
		Poor	Good			
**Gender**						
Female	982	542 (55.2)	440 (44.8)	1	27.43	< 0.001*
Male	585	399 (68.2)	186 (31.8)			
Total	1567	941 (60.1)	626 (39.9)			
**Age group**						
20–29	303	190 (62.7)	113 (37.3)	4	20.68	< 0.001*
30–39	618	408 (66.0)	210 (34.0)			
40–49	367	197 (53.7)	170 (46.3)			
50–59	211	115 (54.5)	96 (45.5)			
60–69	6	2 (33.3)	4 (66.7)			
Total	1505	912 (60.6)	593 (39.4)			
**Years in practice**						
0–2	512	325 (63.5)	187 (36.5)	3	25.94	< 0.001*
3–5	328	217 (66.2)	111 (33.8)			
6–10	263	167 (63.5)	96 (36.5)			
≥ 11	471	239 (50.7)	232 (49.3)			
Total	1574	948 (60.2)	626 (39.8)			
**Education level**						
National diploma	134	67 (50.0)	67 (50.0)	2	6.72	0.035*
Bachelor	1076	663 (61.6)	413 (38.4)			
Masters or Ph.D.	359	217 (60.4)	142 (39.6)			
Total	1569	947 (60.4)	622 (39.6)			
**Designation**						
Nurse	570	291 (51.1)	279 (48.9)	7	59.30	< 0.001*
Medical practitioner^a^	609	422 (69.3)	187 (30.7)			
Pharmacist	145	104 (71.7)	41 (28.3)			
Physiotherapist	120	63 (52.5)	57 (47.5)			
Radiographer	7	5 (71.4)	2 (28.6)			
Medical lab. scientist	108	56 (51.9)	52 (48.1)			
Occupational therapist	10	3 (30.0)	7 (70.0)			
Total	1569	944 (60.2)	625 (39.8)			

^a^Medical practitioner = physicians, surgeons, psychologists, dentists.

*χ^2^-statistic was significant at p < 0.05.

**Table 2:** Participants’ demographic characteristics (n = 1580).

Parameter	n	Quality of care *f* (%)		*df*	χ^2^-statistic	*p*-value
		Poor	Good			
**Appointment**						
Full time	1380	806 (58.4)	574 (41.6)	2	20.35	< 0.001*
Part time	187	140 (74.9)	47 (25.1)			
Casual	10	4 (40.0)	6 (60.0)			
Total	1577	950 (60.2)	627 (39.8)			
**Work schedule**						
PM and call duty	789	498 (63.1)	291 (36.9)	3	10.69	0.014*
Shift duty	468	253 (54.1)	215 (45.9)			
Shift and call duty	30	18 (60.0)	12 (40.0)			
PM	289	180 (62.3)	109 (37.7)			
Total	1576	949 (60.2)	627 (39.8)			
**Work volume**						
< 20 h	67	42 (62.7)	25 (37.3)	3	5.85	0.119
20–40 h	419	266 (63.5)	153 (36.5)			
41–60 h	739	423 (57.2)	317 (42.8)			
> 60 h	350	219 (62.8)	130 (37.2)			
Total	1575	950 (60.3)	625 (39.7)			

*χ^2^-statistic was significant at p < 0.05.

### Levels of care

Participants’ reported quality of care is shown in Tables 3 and 4. On a scale of 0 (never) to 5 (always), the participants reported their levels of person-centred care. Many of the participants (n = 1288, 81.5%) reported that they always or frequently observed progress in their patients, 1267 (80.2%) provided high-quality clinical services, 1340 (84.8%) felt they were compassionate, 1289 (81.6%) involved patients in decisions about their care, but 877 (55.5%) went beyond the normal call of duty to support patients. Responses to items on discordant care (Tables 3 and 4) showed that “always or frequently”, some participants (n = 257, 16.3%) had conflicts with patients, 307 (19.4%) delayed certain patients, 324 (20.5%) treated certain patients with bias, 577 (36.5%) exhibited governmentality, while 271 (17.2%) felt irritable interacting with patients.

**Table 3:** Response distribution on Clinician Quality of Care scale (n = 1566).

Item	Never	Very rarely	Rarely	Occasionally	Very frequent	Always	Mean (median)
Person-centred care	0	1	2	3	4	5	
	*f* (%)	*f* (%)	*f* (%)	*f* (%)	*f* (%)	*f* (%)	
I saw positive progress in my clients/patients	5 (0.3)	6 (0.4)	30 (1.9)	237 (15)	962 (60.9)	326 (20.6)	4.0 (4)
I feel I provided high quality services to clients/patients	2 (0.1)	10 (0.6)	29 (1.8)	258 (16.3)	852 (53.9)	415 (26.3)	4.0 (4)
I felt connected to the clients/ patients I am working with	8 (0.5)	15 (0.9)	64 (4.1)	311 (19.7)	776 (49.1)	392 (24.8)	3.9 (4)
I felt like I was able to really show compassion to a patient	2 (0.1)	9 (0.6)	26 (1.6)	189 (12)	859 (54.4)	481 (30.4)	4.1 (4)
I had space in my schedule to address patient emergencies	16 (1)	25 (1.6)	96 (6.1)	450 (28.5)	655 (41.5)	324 (20.5)	3.7 (4)
I helped a client/patient develop a safety plan to address potentially harmful behaviour or situations	12 (0.8)	33 (2.1)	110 (7)	456 (28.9)	664 (42)	291 (18.4)	3.7 (4)
I was able to support a client’s/patient’s action step toward a personal goal	21 (1.3)	32 (2.0)	117 (7.4)	486 (30.8)	645 (40.8)	265 (16.8)	3.6 (4)
I involved clients/patients in decisions about their care	9 (0.6)	9 (0.6)	46 (2.9)	213 (13.5)	744 (47.1)	545 (34.5)	4.1 (4)
I spent extra time with a client/patient who needed support	6 (0.4)	18 (1.1)	50 (3.2)	397 (25.1)	695 (44)	401 (25.4)	3.9 (4)
I was able to come up with a creative intervention to support a client/patient	16 (1.0)	20 (1.3)	94 (5.9)	529 (33.5)	651 (41.2)	256 (16.2)	3.6 (4)
I went “above and beyond the normal call of duty” to support a client/patient	10 (0.6)	28 (1.8)	106 (6.7)	545 (34.5)	611 (38.7)	266 (16.8)	3.6 (4)
I met my daily productivity expectations	3 (0.2)	22 (1.4)	72 (4.6)	420 (26.6)	773 (48.9)	276 (17.5)	3.8 (4)

These were responses to the instruction “Please, indicate how frequently each item had occurred in the past 6 months” Where 0 = never, 1 = very rarely, 2 = rarely, 3 = occasionally, 4 = very frequently, 5 = always.

Total respondent’s score was converted to percentages (expected range, 0–110 X 0.909 = 0–100%).

The present respondents’ scores ranged from 10.91 to 100%, mean = 70.14, median = 70.91, and SD = 12.76.

**Table 4:** Response distribution on Clinician Quality of Care scale (n = 1566).

Item	Never	Very rarely	Rarely	Occasionally	Very frequent	Always	Mean (median)
Discordant care	0	1	2	3	4	5	
	*f* (%)	*f* (%)	*f* (%)	*f* (%)	*f* (%)	*f* (%)	
I had conflicts with clients/patients	338 (21.4)	501 (31.7)	279 (17.7)	191 (12.1)	180 (11.4)	77 (4.9)	1.8 (1)*
I made minor mistakes in my work (not likely to affect clients/patients)	148 (9.4)	580 (36.7)	330 (20.9)	263 (16.6)	190 (12)	55 (3.5)	2.0 (2)*
I took a long time responding to certain client/patient requests	219 (13.9)	376 (23.8)	306 (19.4)	358 (22.7)	224 (14.2)	83 (5.3)	2.2 (2)*
I treated clients/patients differently because they are my favourites	591 (37.4)	267 (16.9)	211 (13.4)	173 (10.9)	155 (9.8)	169 (10.7)	1.7 (1)*
I was usually directive with clients/patients (telling them what to do)	159 (10.1)	235 (14.9)	239 (15.1)	356 (22.5)	384 (24.3)	193 (12.2)	2.7 (3)*
I was irritable interacting with clients/patients	605 (38.3)	368 (23.3)	195 (12.3)	127 (8.0)	121 (7.7)	150 (9.5)	1.5 (1)*
I missed appointments or meetings with clients/patients	529 (33.5)	407 (25.8)	212 (13.4)	138 (8.7)	138 (8.7)	142 (9)	1.6 (1)*
I missed deadlines at work	439 (27.8)	435 (27.5)	214 (13.5)	196 (12.4)	146 (9.2)	136 (8.6)	1.7 (1)*
I had significant distractions in my work with clients/patients	381 (24.1)	422 (26.7)	267 (16.9)	226 (14.3)	156 (9.9)	114 (7.2)	1.8 (1)*
I was late for work	248 (15.7)	465 (29.4)	296 (18.7)	316 (20)	176 (11.1)	65 (4.1)	1.9 (2)*

These were responses to the instruction “Please, indicate how frequently each item had occurred in the past 6 months” Where 0 = never, 1 = very rarely, 2 = rarely, 3 = occasionally, 4 = very frequently, 5 = always.

Total respondent’s score was converted to percentages (expected range, 0–110 X 0.909 = 0–100%).

The present respondents’ scores ranged from 10.91 to 100%, mean = 70.14, median = 70.91, and SD = 12.76.

*Items were reverse coded during computation and inferential analyses.

### Correlation and differences in personal well-being, health, and quality of work-life across levels of care

Table 5 shows that participants who reported lower health-related quality of life had higher quality of care scores (r = − 0.104, p < 0.001). However, there was a positive correlation between quality of care and personal well-being (r = 0.153, p < 0.001), and quality of work-life (r = 0.201, p < 0.001). However, when the quality-of-care scores were dichotomised as poor (< 75%) and good (≥ 75%), there was no significant difference in the mean quality of life between those that rendered poor and good quality of care, t = 1.102, p = 0.271 (Table 6). However, HCPs that delivered poor quality of care had significantly lower personal well-being index (t = − 6.396, p < 0.001), and quality of work-life (t = − 8.575, p < 0.001).

**Table 5:** Pearson’s Correlation: among respondents’ quality of life, well-being, quality of work-life, quality of care.

Indexes	Personal well-being	Quality of work-life	Quality of care
	*r*-statistic (N) p-value	*r*-statistic (N) p-value	*r*-statistic (N) p-value
WHO-quality of life	0.266 (1575)	0.212 (1570)	− 0.104 (1562)
	< 0.001*	< 0.001*	< 0.001*
Personal well-being	–	0.521 (1572)	0.153 (1565)
		< 0.001*	< 0.001*
Quality of work-life			0.201 (1561)
	–	–	< 0.001*

*Pearson’s Correlation Coefficient (r) was significant at p < 0.05 (2-tailed test).

**Table 6:** Respondents’ quality of life, well-being, quality of work-life by quality of care.

Parameter	Clinician quality of care		t-value	p-value
	Poor Mean (SD)	Good Mean (SD)		
Quality of life	62.36 (19.49)	61.11 (23.77)	1.102	0.271
Personal well-being	69.74 (14.30)	74.50 (14.71)	− 6.396	< 0.001*
Quality of work-Life	63.92 (9.80)	68.46 (10.99)	− 8.575	< 0.001*

*Independent samples t-test was significant at p < 0.05 (2-tailed test).

### Confirmatory factor analysis and structural equation model

**Figure 1:**
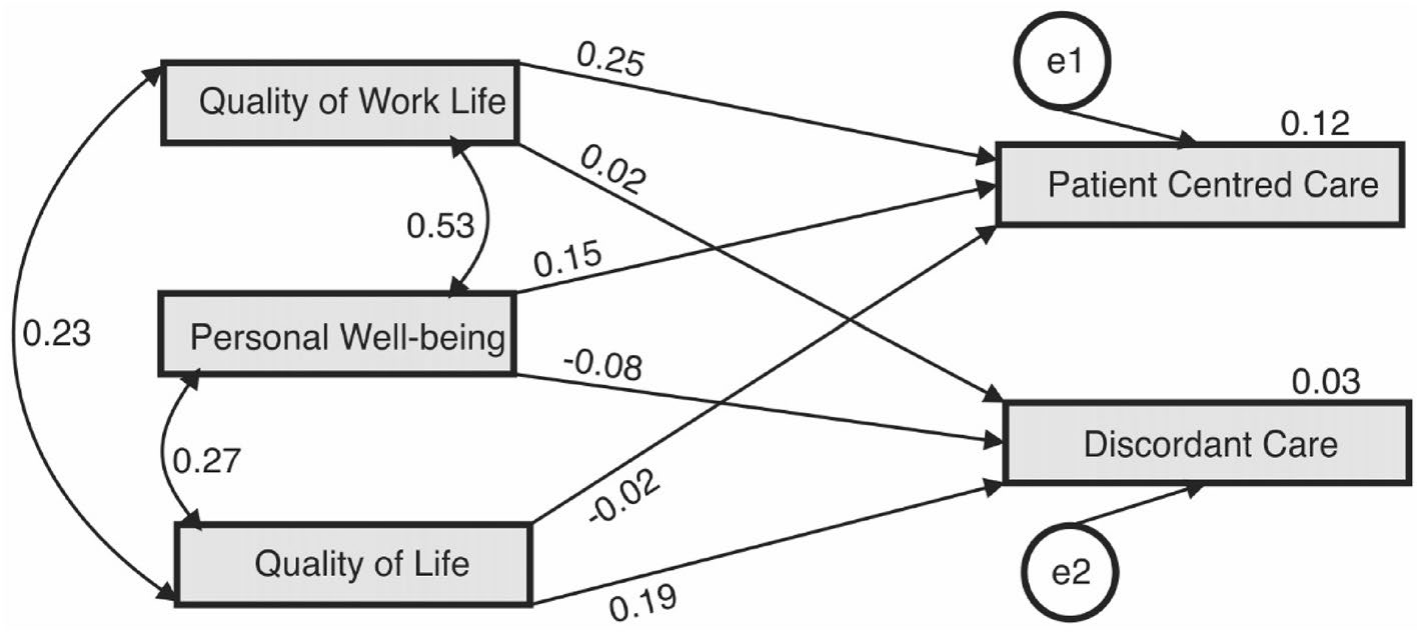
Path diagram showing the standardised regression weight of association between the constructs (created by authors). This figure shows the structural equation diagram for path analysis of associations between person-centred and discordant care with quality of life, personal well-being index and quality of work-life scores.

The confirmatory factor analysis showed that the aggregate-questionnaire items belong to five distinct domains: QoL-5 loaded 5/5 items (at factor 5), PWI loaded 9/8 items (at factor 3) including the tenth item of the WRQoL questionnaire, WRQoL loaded 15/24 items (at factors 2) because items with variances less than 0.45 were supressed, person-centred QoC = 12/12 items (at factors 4), and discordant QoC loaded 10/10 (at factor 1) including the ninth item of the WRQoL questionnaire. The cumulative variances explained was 44.23%: discordant QoC = 10.74%, WRQoL = 10.73%, PWI = 9.03%, person-centred QoC = 8.01%, and WHO-QoL = 5.72%. Chi-square goodness of fit for CFA was χ^2^(1426) = 6175.22, p < 0.001. Figure 1 shows the structural equation diagram for path analysis of associations between person-centred and discordant care with QoL, PWI, and WRQoL scores. There were significant associations among person-centred care and WRQoL (β = 0.25, p < 0.001), person-centred care and PWI (β = 1.5, p < 0.001), and discordant care and PWI (β = -0.08, p = 0.01). All the covariances paths had significant associations (p < 0.001). Although the sample size was large making Chi-square goodness of fit to be significant (χ^2^[1, N = 1558] 5.384, p = 0.02), the model modestly fitted the data, CFI = 0.995, TLI = 0.951, and RMSEA = 0.053.

## Discussion

Health care professionals work in environments that are biologically hazardous with well-documented physical and psychological demands^32^. The perceived influence of HCPs’ well-being at work on the quality of care rendered to patients was investigated. In this present study, we observed that the majority, that is, six (6) out of every 10 HCPs were females. This finding corroborates the global healthcare gender composition estimate as reported by Langer et al.^33^ that women comprise seven out of ten health and social care workers and contribute US$ 3 trillion annually to global health. The medical practitioners and nurses had 75% of the healthcare workforce involved in this research. A few reasons for the preponderance of doctors and nurses among HCPs may include the awareness of the profession, the profession’s prestige, the availability of such profession as a course of study in the country’s institutions, the employment rate in the country, the remuneration, among others^3^. The National Human Resources for Health Strategic Plan^34^ reports that a nurse: population ratio of 100:100,000 as compared to a doctor: population ratio of 30:100,000, a pharmacist: population ratio of 11:100,000 and a physiotherapist: population ratio of 0.62:100,000 in Nigeria. This uneven and low distribution of HCPs in the Nigerian health workforce is sometimes responsible for nurses having to perform the role of doctors in some health institutions in the country. This has been shown to be responsible for the low job satisfaction, heightened work stress, frustration, and burnout experienced by HCPs in Nigeria^8^.

Findings from this study showed that in the person-centred care domain, 8 out of every 10 HCPs reported good practice while less than 2 out of every 10 HCPs reported good practice on discordant care in this study. Also, majority reported poor QoC and this may be associated with the fact that the average HCP in Nigeria work with obsolete tools, are not well motivated and the presence of skewed health governance^5^. It has been reported that the deficits in quality of care appear to be more pronounced in low- and middle-income countries (LMICs) where an estimated 8 million lives are lost annually to poor quality of care^35,36^. Odunaiya et al.^37^, in a study among HCPs in Nigeria, reported that the quality of care for cardiac patients in Nigeria was sub-optimal, as perceived by HCPs and this was attributed to poor staff strength, inadequate opportunities for further training, poor infrastructural planning and procurement, poor adherence to treatment guidelines, lack of a system for internal quality assurance, poor inter-professional collaboration and other administrative issues such as patient waiting time, patient recordkeeping and retrieval. Odusola et al.^38^ identified high staff workload and administrative challenges as inhibitors of quality medical care for hypertensive patients in primary health centres in Nigeria. Ephraim-Emmanuel^39^ also opined that inequitable distribution of the limited available resources and personnel in the Nigerian healthcare system has persistently affected the quality of care. All these concerns must be addressed if the quality of healthcare in Nigeria is to improve.

In this study, we found that the HCPs who reported to have delivered person-centred model of care did that at the expense of their quality of life, while those who had good personal well-being and quality work-life provided person-centred care. This finding complemented that of the correlation analysis in this study which showed a negative correlation between clinicians’ health-related quality of life and their quality of care, but positive correlation between their personal well-being, quality of work-life and quality of care. There is a need to balance HCPs’ quality of life and the desire for person-centred care delivery. To achieve this, their well-being and quality of work-life should be improved. There was a significant negative correlation observed between HCPs quality of life and the quality of care rendered by these HCPs, implying that HCPs with low quality of life claimed to have rendered better quality of care. In previous studies, a direct relationship was reported between quality of care and health-related quality of life using the outcome method for assessing quality of care^40,41^. This appears to be plausible as the outcome method of assessing quality of care is expected to be more objective than the process method, although, none of the results from these methods should be used in isolation^42^. Also, quality of life is a measure of dysfunction in well-being buttressing the claims that this set of HCPs have certain derangement in health which may condition them to be more empathetic, thus rendering better quality of care to patients. However, there was a significant positive correlation observed amongst HCPs personal well-being index and quality of care rendered. This appears plausible as personal well-being is a measure of a personal aspect of the quality of life that describes good, satisfactory, and desirable state of personal existence or life^43^. Therefore, HCPs who reported better well-being are expected to render good quality of care to patients.

On well-being, the outcome of our research is in keeping with those of other researchers^44,45^ who reported that good well-being at work will improve the quality of care rendered by HCPs. Findings from the present study are also consistent with previous studies which have reported a relationship between the well-being of HCPs and the quality and safety of patient’s care^46–49^. West et al.^46^ and Johnson et al.^48^ opined that the direction of the well-being of HCPs and the quality of patient’s care can be described to operate as a feedback loop. Improved well-being of HCPs may lead to the provision of high-quality care while poor well-being may pose a hindrance to the delivery of quality care^46,48^. The hospital environment in the context as reported previously does not provide optimal well-being and quality of work life for health care professionals^24^, therefore employers of labour can improve productivity and service delivery by actively bringing up policies that will improve the work environment of HCPs. This in turn will help to improve the HCPs’ well-being and the quality of care delivered to patients.

### Limitations

This study has provided useful information on the well-being, and quality of work-life of HCPs and its impact on quality of care, however, the study did not consider other factors such as family life and personal factors that could impact the well-being and quality of work-life of the HCPs.

## Conclusion

We concluded that HCPs’ well-being and quality of work-life are important factors that influence the quality of care rendered to patients. The hospital management and policymakers should ensure improved work-related factors to improve the well-being of HCPs and enhance the quality of care given to patients. The findings of this study could be used to establish policies and initiatives to promote the well-being and quality of work-life of Nigerian HCPs. When HCPs' well-being and quality of work-life are improved, the quality of care they deliver to patients should also improve.

## Data Availability

The data that support the findings from this study are available from the corresponding author on reasonable request.
